# Fluoride Content of Matcha Tea Depending on Leaf Harvest Time and Brewing Conditions

**DOI:** 10.3390/nu14122550

**Published:** 2022-06-20

**Authors:** Karolina Jakubczyk, Alicja Ligenza, Izabela Gutowska, Katarzyna Janda-Milczarek

**Affiliations:** 1 Department of Human Nutrition and Metabolomics, Pomeranian Medical University in Szczecin, 24 Broniewskiego Street, 71-460 Szczecin, Poland; karolina.jakubczyk@pum.edu.pl (K.J.); alicjalig@gmail.com (A.L.); katarzyna.janda.milczarek@pum.edu.pl (K.J.-M.); 2 Department of Medical Chemistry, Pomeranian Medical University in Szczecin, 72 Powstancow Wlkp Street, 71-460 Szczecin, Poland

**Keywords:** matcha, green tea, fluoride, powder

## Abstract

Matcha, or powdered green tea (*Camellia sinensis*) of the Tencha type, is popular all around the world, and its consumption continues to rise. Because of its unique cultivation method, it is rich in phytochemicals and has many health-promoting properties; it contains high concentrations of polyphenols, theanine and chlorophyll. Tea, and by extension matcha, contains numerous minerals, one of which is fluorine. Under physiological conditions, this mineral plays a significant role in hard tissue mineralisation processes. However, even in low concentrations, with prolonged exposure, fluoride can accumulate in the body, leading to a number of harmful effects. The aim of this study was to evaluate, for the first time, the fluoride content of the matcha infusions from different harvests, brewed using water at different temperatures (25 °C, 70 °C, 80 °C and 90 °C). The content of fluoride ions was measured by the potentiometric method. The fluoride content ranged from 3.36 to 4.03 mg/L and was dependent on both the leaf harvest time and brewing temperature. The concentration of this mineral in the dry powder ranged from 118.39 to 121.65 mg/kg. Irrespective of the water temperature or harvest time, matcha was found to have a high fluoride content, with particularly high concentrations being noted in the powder itself.

## 1. Introduction

Tea (*Camellia sinensis*), with its health-promoting as well as organoleptic properties, is known and appreciated all over the world, and tea products are widely available in the market. Three main variants can be found in stores: loose leaf tea, tea bags, and powder [1]. One variety of green tea that is growing in popularity is matcha green tea powder (*Camellia sinensis*) [2,3,4]. It is grown mainly in Japan and, increasingly, also in China and Korea. It is appreciated for its distinctive flavour and aroma, as well as its numerous health-promoting benefits [5,6]. This tea variety is particularly rich in antioxidants, thanks to its unique, historic cultivation method [7,8]. The tea bushes are covered with bamboo mats which provide shade [1]. Shading from excessive direct sunlight enables the plant to produce bioactive compounds, including chlorophyll and l-theanine. As an additional advantage, this cultivation method is responsible for the unique sensory properties of matcha tea, such as the vibrant colour, aroma and umami taste, which is related to the lower content of catechins and the high content of caffeine, theanine and chlorophyll in the product [9]. The traditional method of growing tea in full sun produces a higher catechin content, imparting a bitter taste to infusions, which often puts consumers off [10]. Matcha, on the other hand, is highly valued for its quality and regarded as the most aromatic green tea variety [3,9,11].

The health benefits of drinking green tea are related to the presence of antioxidants, notably the wide range of polyphenols: the flavonols, flavandiols and phenolic acids, which account for up to 30% of the dry weight [12]. Due to the antioxidant, antiviral, anti-inflammatory, immune-stimulating and detoxifying properties of green tea, its consumption has been associated with a reduced risk of cardiovascular disease and cancer [13,14,15,16]. The quantities of the health-promoting substances contained in tea beverages depend on the type of tea, portion size, brewing temperature and time [17].

Matcha green tea contains numerous minerals, and one of these is fluorine [18]. Under physiological conditions, fluorine plays a significant role in hard tissue mineralisation processes [19,20,21]. However, there is a thin line between the amount of fluorine that is beneficial for metabolic processes and a harmful dose. In addition, with prolonged fluoride exposure, even low concentrations can accumulate in the body [22]. The mechanism behind fluorine toxicity involves the enhanced production of ROS (oxygen free radicals), increased lipid peroxidation and the altered activity of many enzymes [19,20,22]. This can disrupt the normal functioning of the body and lead to fluorosis, a disease that primarily affects the hard tissues of the body. Additionally, fluoride easily crosses cell membranes, entering soft tissues, which promotes its accumulation [19,20,21,22,23,24].

Despite its long tradition of use, matcha tea is a relatively new addition to the market and has not been extensively studied. Consequently, the aim of this study is to examine, for the first time, the fluoride content of the matcha powder itself and of the tea with different harvest times (traditional and daily) and prepared at different temperatures.

## 2. Materials and Methods

### 2.1. Plant Material

The material studied consisted of two types of high-quality, organic Japanese green matcha tea (*Camellia sinensis*), made from the leaves of Tencha, originating from the Uji region of Japan in the Kyoto prefecture. The first type, Traditional matcha, comes from the first and second harvest of the leaves, whereas the second type, Everyday matcha, comes from the second and third harvest. The green tea harvest begins at the end of April. The first harvest lasts until the end of May; it is ichibancha—literally, the “first tea”. After the tea leaves are picked, new tea leaves appear in their place, which are harvested at the turn of June and July—the second harvest. The third harvest of the year takes place in August. Tea from each subsequent harvest is a bit weaker, poorer in colour and taste. During production, the resource was subjected to drying and grinding and did not require another homogenization before study.

### 2.2. Preparation of Infusion

An amount of 1.75 g of a plant material sample was transferred to a conical flask, to which 100.0 mL of distilled water was added at a given temperature (25 °C, 70 °C, 80 °C, or 90 °C) [9]. The flask with the infusion was closed and rotated at a speed of 180 rpm (Brunswick model EXCELLA E24, Brunswick, Canada) for 10 min. After brewing, the plant parts were separated from the infusion through filtration. All the infusions were performed in three repetitions.

### 2.3. Determination of the Fluoride Content in Prepared Samples

Sample levels of F^−^ were determined using a potentiometricion-selective electrode (Thermo Scientific Orion, Waltham, MA, USA) according to the works of Gutowska et al. and Łukomska [9,12]. An amount of 0.5 mL of the matcha infusion (TISAB II solution) was added to the test tube, and then the potential was measured for 5 min. After saving the result, an appropriate standard was added to the sample and re-measured for the same amount of time. The fluoride content in the samples was calculated based on the difference in the potentials measured in each sample and the concentration level of the added standard. The electrode was calibrated using standard solutions.

### 2.4. Statistical Analysis

In all the experiments, three samples were analysed, and all the assays were carried out at least in triplicate. The statistical analysis was performed using Stat Soft Statistica 13.0 and Microsoft Excel 2017. The results were expressed as the mean values and standard deviation (SD). To assess the differences between the examined parameters’ one-way analysis of variance (ANOVA), a Tukey post hoc test was used. The Pearson test was used to calculate the correlation coefficient. Differences were considered significant at *p* ≤ 0.05. To control for type I errors, the false discovery rate (FDR) approach was used. The calculations were performed using the *p*. adjust function of the stats package in R (R Foundation for Statistical Computing, Vienna, Austria, https://cran.r-project.org). (accessed on 10 January 2022).

## 3. Results and Discussion

The main sources of fluoride intake for humans are water, air pollutants, dental preparations and food. The fluoride content of many teas, especially green tea, is very high [25]. Given the side effects of excess fluoride and its deposition in the body, it is necessary to pay attention to its intake from food and the environment to avoid the consequences of overdose.

The fluoride content found for traditional matcha powder was 118.39 ± 0.10 mg/kg, while that for daily matcha powder was slightly higher at 121.65 ± 2.39 mg/kg; this difference was found to be statistically significant (FDR *p* < 0.05) (Table 1).

The fluoride content in matcha infusions ranged from 3.36 to 4.03 mg/L and was dependent on both the leaf harvest time and brewing temperature (Table 2). For both daily and traditional matcha, the lowest values were recorded at 25 °C and the highest at 70 °C and 90 °C. A statistical analysis of the data revealed that, for infusions made with both matcha types, significant differences in the fluoride content were observed for those prepared with water at 25 °C vs. 70 °C and for those prepared with water at 25 °C vs. 90 °C. There were no significant statistical differences in fluoride content between the infusions of traditional and daily matcha made at the same temperature.

There was also a statistically significant, moderate positive correlation between the fluoride concentration and the temperature (0.675—daily matcha; 0.645—traditional matcha). It can therefore be concluded that, the higher the water temperature used to prepare the brew, the higher the concentration of fluoride in the beverage.

The Recommended Dietary Allowance (RDA) for fluoride is 3 mg/day (Food and Nutrition Board, Institute of Medicine), which means that one cup of either traditional or daily matcha would cover 14–16.7% of the requirement for that mineral. However, with three cups, this can go up to as much as 66.8% of the recommended daily intake (Table 3 and Table 4).

Fluoride concentration levels in teas ranged from 3.36 to 4.03 mg/L. The highest level was found in daily matcha tea and the lowest in traditional matcha tea. Most people drink between one and three cups of tea and similar beverages, including green tea, per day. According to our findings, a 125 mL cup of traditional matcha infusion contains 0.42, 0.50, 0.47 and 0.49 mg fluoride for beverages made at 25 °C, 70 °C, 80 °C and 90 °C, respectively; a 125 mL cup of daily matcha infusion contains 0.42, 0.50, 0.46 and 0.5 mg fluoride for beverages made at 25 °C, 70 °C, 80 °Cand 90 °C, respectively.

According to the EFSA (European Food Safety Authority), an adequate intake of fluoride is defined as > 0.05 mg per kg body weight. For example, for a person weighing 60 kg, the norm is 3 mg, for 70 kg, it is 3.5 mg, and for 80 kg, the norm is 4 mg. On the other hand, the recommended dietary allowance (RDA) for fluoride is 3 mg/day (Food and Nutrition Board, Institute of Medicine), so one cup of either traditional or daily matcha would cover 14–16.7% of the requirement for that mineral, while for three cups, this amount can go up to as much as 66.8%.

The fluoride content in beverages may be affected by a range of different factors; the authors cite differences in the topographical location of the raw material; differences in the mineral content of the soil, water and atmosphere; variation in the use of agrochemicals and variation in the processes of tea fermentation or beverage brewing. A significant correlation was identified between the fluoride content in the water used in the preparation of the beverages and that in the final product [26]. Therefore, both the quality of the source material and that of the water used in the preparation of the beverage affect the final outcome. It is worth adding that distilled water was used in the present study, while the tap water used in domestic environments and in the food industry would provide an additional source of fluoride. Janda-Milczarek et al. [25] suggest that the process of extracting fluoride from the tea into the infusion is dependent on a number of factors. Undeniably, one of the primary factors affecting the extraction process is the concentration level used. The number of infusions made from the same material is another important factor. Łukomska et al. [27] and Fung et al. [28] noted that the fluoride content of infusions decreased with each successive infusion. It is quite common for green tea to be brewed multiple times, and matcha tea in its powdered form dissolves in water very well. Emekli-Alturfan et al. [29] observed a minor positive effect of the brewing time on the fluoride content of herbal and fruit infusions, which was also confirmed in the studies by Chan and Koh [30] and Fung et al. [28]. Malinowska et al. [31] noted that the effect of the brewing time was significantly greater for black tea than for other types of tea. Chan and Koh [30] concluded that the fluoride content of tea infusions is influenced by the brand of tea, its type, and the presence of caffeine, with a higher content being found in decaffeinated teas. Gupta and Sandesh [32], Wolska et al. [33] and Janda et al. [25] also demonstrated that the fluoride content of the brew is significantly affected by the form of the source material and the brewing method.

In our study, there were no statistically significant differences in the fluoride concentration levels between the infusions prepared with daily matcha and traditional matcha at the same temperature. However, it is worth noting that the traditional matcha came from the first and second harvest, while the daily matcha came from the second and third harvest. Hence, the harvest time was similar, and in our case, did not affect the concentration and accumulation of the mineral compounds in leaves. The absence of statistically significant differences may also be related to having the same growing conditions and similar environmental factors. On the other hand, the fluoride concentration was significantly affected by the temperature of the water used to prepare the infusion. In our study, statistically significant differences were noted between infusions prepared using water at room temperature and those made at 70 °C and 90 °C for both traditional and daily matcha. A positive correlation was also observed between the fluoride concentration and the water temperature (*p* < 0.05). Thus, the use of higher temperatures is associated with an increase in the fluoride content of the infusion, which may lead to exceeding the safety limits for consumption, bearing in mind how many food products contain this mineral. Few scientific reports confirm that the positive correlation between the concentration of fluoride and the temperature of the infusion may be associated with an increase in the release of fluoride from dried plants, which may be related to the loosening of the raw material and an increase in kinetic energy [32,33,34]. In an article by P. Gupta and N. Sandesh, the influence of various brewing methods on the concentration of fluorides in 16 teas was examined. Tea infusions were prepared by three different methods, i.e., without boiling with water, after boiling with water, and after the addition of milk and sugar to boiling water. In the first method using water at room temperature, a significantly lower concentration (1.437 mg/L) was recorded than in the second method using hot water (3.375 mg/L). The authors concluded that the low concentration of fluoride in the infusion of tea before boiling the water, regardless of the form of the tea, may be due to the fact that these ions are released in small amounts at room temperature, and they may take longer to be released in the tea infusion. The high concentration of fluoride in the tea infusion prepared after boiling the water, regardless of the form of the tea, may be due to the fact that the increase in temperature favours the release of more of the tea in the tea infusion [32]. In the case of our results, these differences were smaller, which is related to the strong fragmentation of the raw material and easier ion release, even at low temperatures. Dębia et al. [35] investigated the effect of the temperature of water (25 °C, 70 °C, 80 °C and 90 °C) used for the preparation of gout (*Aegopodium podagraria*) infusions on the content of fluoride. Different morphological parts of the plant were used for the study. A statistically significant, positive correlation (r = 0.74) was found for infusions from flowers. The fluoride content increased with the rise in water temperature used for the preparation of the solutions. The highest value (0.25 mg/L) proved to be significantly higher than the lowest, achieved at 25 °C (0.14 mg/L), and the value at 80 °C (0.18 mg/L). The fluoride content achieved at 70 °C (0.17 mg/L) was significantly higher than that at 25 °C (0.14 mg/L) and lower than the highest value, which was observed for the infusions prepared at 90 °C (0.25 mg/L). Importantly, the bioavailability of fluoride from liquids is much higher than that from solid foods, additionally increasing exposure to the mineral [36]. On the other hand, it is worth noting that higher water temperature is also associated with an increase in the powerful antioxidant properties of matcha infusions [9]. Therefore, it is advisable to exercise moderation in the number of cups of matcha consumed, so as to provide necessary antioxidants, on the one hand, and, on the other, maintain a safe level of fluoride intake.

The findings from our study on the fluoride content in tea infusions are consistent with those from other authors. While numerous scientific reports confirm that tea is a major dietary source of fluoride, there have been few studies on the content of fluoride ions in matcha green tea. Regelson et al. [37] tested many tea infusions for fluoride content, including those from matcha (Mighty Leaf (Emeryville, CA, USA), Celestial Seasonings (Boulder, CO, USA), and Matcha Love) (Brooklyn, NY, USA)(. All the tested samples contained fluoride in amounts ranging from 0.521 to 6.082 mg/L, which is consistent with the present findings. Matcha green tea powder had the highest concentration of fluoride, but it is important to note that it was brewed with boiling water [37]. In contrast, in our study, the fluoride content was examined in matcha green tea made with water at different temperatures; we also accounted for the difference in harvest times. Emekli-Alturfan et al. [29] and Malinowska et al. [31] tested 1.5–2% infusions of black tea. Their fluoride content amounted to 1.21–3.56 µg/mL, 0.57–3.53 mg/L and 0.32–6.87 mg/L. In turn, according to Malinowska et al. [31], the content of this mineral was: for green tea, 0.59–2.52 mg/L; green tea with additives, 0.08–1.7 mg/L; oolong or Pu-erh tea, 0.39–2.85 mg/L; white tea, 0.37–0.69 mg/L; herbal tea, 0.02–0.14 mg/L. These results are similar to the findings from the present study. Satou et al. [38] conducted a study where they examined the fluoride content of dry tea and tea infusions, including green tea. The results for green tea showed the highest fluoride concentrations: 0.26–4.09 mg/L and 21.91–83.68 mg/kg. The researchers concluded that the habitual consumption of certain foods, especially green tea, requires the risk management of dental fluorosis [38].

Matcha tea powder is increasingly used in cooking; it can be added to baked goods, desserts, dairy products and many dishes. Based on our study, 1 tablespoon, or 10 g of daily matcha powder containing 1.22 mg of fluoride, covers 44.55% of fluoride requirement, while the same amount of traditional matcha (1.18 mg of fluoride) covers 39.46% of fluoride requirement. Štepec et al. [39] analysed the fluoride content in eight samples of superfoods, including dry matcha tea *(Camellia sinensis* L). The highest fluoride content was found in matcha, amounting to 373 μg/g. Unlike in our study, where the fluoride content of dry traditional matcha was 118.39 mg/kg and that of daily matcha was 121.65 mg/kg, the tea studied by the researchers contained only 0.373 mg/kg of fluoride. Still, the authors expressed their concern about the dangerously high fluoride content of matcha [39]. It should be noted that this is a significantly lower level of the mineral compared to the present study, which may be attributable to the origin of the tea. Łukomska et al. demonstrated that the country of origin can significantly affect the fluoride concentration in yerba mate. Teas originating from volcanic areas contained higher concentrations of this element. Thus, growing conditions, including environmental factors and soil composition, significantly affect this parameter.

It is worth emphasising that there is little research dedicated to analysing the composition of matcha and/or other trendy food products in terms of safety, and this area of scientific research should be expanded.

## 4. Conclusions

Our study showed that the harvesting date for matcha tea leaves was shown to have a significant effect on the fluoride content of the dried tea (traditional vs. daily). Matcha daily (from the second and third harvest) had a significantly higher concentration of fluoride. In the case of matcha tea, the temperature of the water used to prepare the infusions also had a significant effect on the fluoride content of the beverage. An analysis of the results showed that the higher the water temperature, the higher the fluoride content in the tea.

However, matcha tea, irrespective of the harvest date or brewing temperature, is a major source of fluoride in the human diet. One litre of this beverage can contain approximately 4 mg of fluoride, providing the total daily requirement for this mineral. The lowest fluoride content was found in the beverage made at 25 °C, entailing the safest dose for humans, which this study brings to light for the first time. Matcha powder, which is increasingly popular as an ingredient in various dishes, contains a very high level of this mineral (120 mg/kg). Despite its valuable health-promoting properties and the abundance of phytochemicals, it is important to control the amount of matcha in one’s daily diet and to take into account other sources of fluoride.

## Figures and Tables

**Table 1:** The fluoride content in matcha powder (traditional, daily) * FDR *p* < 0.05 between the types of matcha powder (traditional, daily).

Fluoride Content in Matcha Powder [mg/kg]					
Traditional			Daily		
118.39 *	**±**	**0.10**	121.65 *	±	2.39

**Table 2:** The fluoride content in tea matcha (traditional, daily) prepared at various temperatures. * FDR *p* < 0.05 between the types of matcha (traditional, daily) prepared at various temperatures.

	Fluoride Content in Matcha Tea [mg/L]					
Temp.	Traditional			Daily		
25 °C ^A^	3.36 *^,B,D^	±	0.11	3.38 *^,B,D^	±	0.22
70 °C ^B^	3.96 *^,A^	±	0.26	4.03 *^,A^	±	0.24
80 °C ^C^	3.77	±	0.02	3.67	±	0.33
90 °C ^D^	3.95 *^,A^	±	0.62	4.00 *^,A^	±	0.05

Data represent the mean values ± standard deviations of the three technical replicates. Different letters (A–D) in superscripts represent statistically significant differences in nutrient (*p* ≤ 0.05).

**Table 3:** The concentration of fluoride in traditional matcha infusions depending on the water temperature and the percentage of the Recommended Dietary Allowance (RDA) for the diet.

Temp.	Traditional Matcha Tea Cups Per Day (1 cup = 125 mL)					
	Fluoride Content [mg]			Recommended Dietary Allowances (RDA) [%]		
	1	2	3	1	2	3
25 °C	0.42	0.84	1.26	14.0	28.0	42.0
70 °C	0.50	1.0	1.5	16.7	33.4	66.8
80 °C	0.47	0.94	1.41	15.7	31.4	47.5
90 °C	0.49	0.98	1.47	16.3	32.6	48.9

**Table 4:** The concentration of fluoride in daily matcha infusions depending on the water temperature and the percentage of the Recommended Dietary Allowance (RDA) for the diet.

Temp.	Daily Matcha Tea Cups Per Day (1 cup = 125 mL)					
	Fluoride Content [mg]			Recommended Dietary Allowances (RDA) [%]		
	1	2	3	1	2	3
25 °C	0.42	0.84	1.26	14.0	28.0	42.0
70 °C	0.50	1.0	1.5	16.7	33.4	66.8
80 °C	0.46	0.92	1.38	15.3	31.0	45.9
90 °C	0.50	1.0	1.5	16.7	33.4	66.8
